# Supplementary figures and images for: The crystal structure of Staufen1 in complex with a physiological RNA sheds light on substrate selectivity
Source: Life Sci Alliance. 2018 Oct 18;1(5):e201800187. doi: 10.26508/lsa.201800187 (PMC6238398; doi:10.26508/lsa.201800187)

anti\_GFP

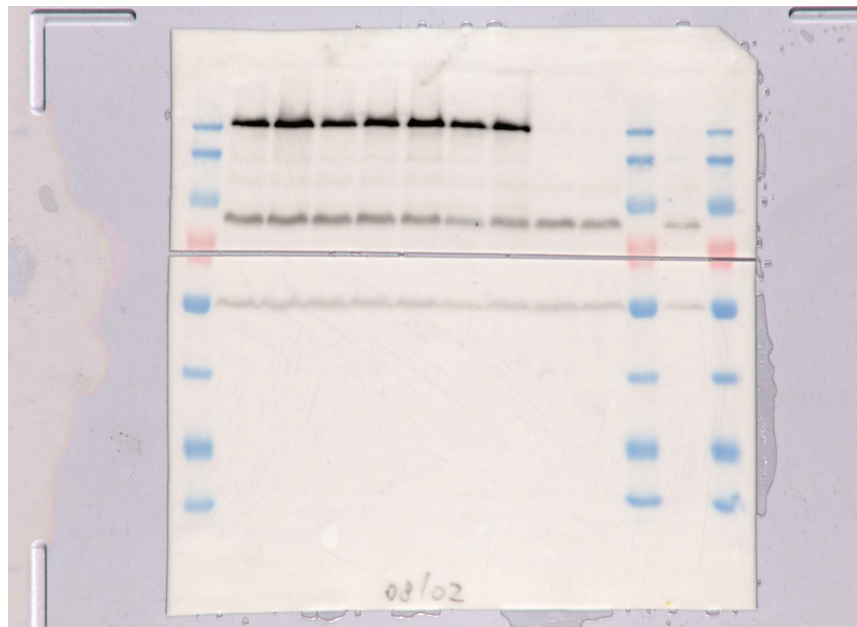

anti\_Tubulin

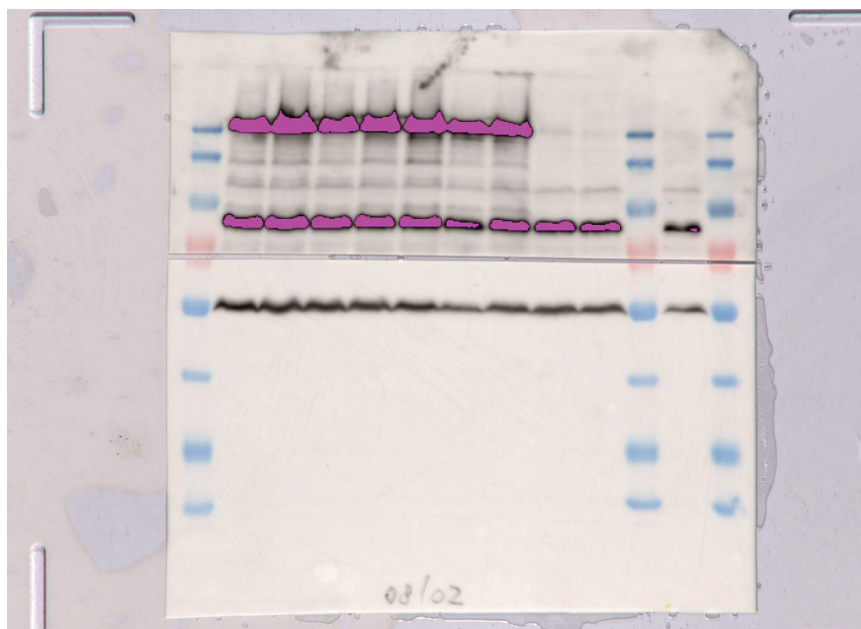

anti\_Stau

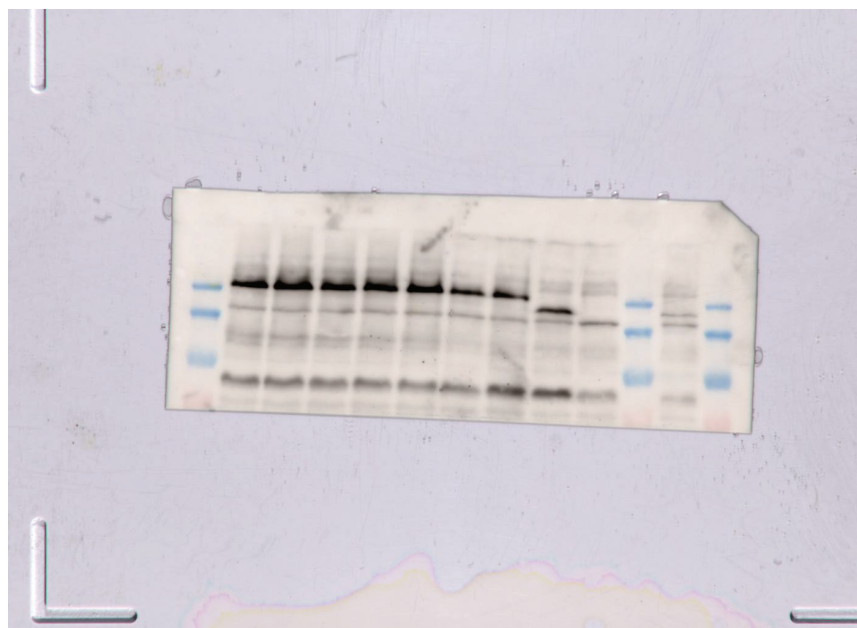

Supplement: Supplementary file 1 [file LSA-2018-00187_SdataF7.pdf]
